# Comparative Analysis of Machine Learning Models for Predicting the Mechanical Behavior of Bio-Based Cellular Composite Sandwich Structures

**DOI:** 10.3390/ma17143493

**Published:** 2024-07-15

**Authors:** Danial Sheini Dashtgoli, Seyedahmad Taghizadeh, Lorenzo Macconi, Franco Concli

**Affiliations:** 1 Department of Mathematics, Informatics and Geosciences, University of Trieste, 34128 Trieste, Italy; danial.sheinidashtgoli@phd.units.it; 2 National Institute of Oceanography and Applied Geophysics-OGS, 34010 Sgonico, Italy; 3 Faculty of Engineering, Free University of Bozen-Bolzano, Piazza Universität 5, 39100 Bolzano, Italylorenzo.maccioni@unibz.it (L.M.)

**Keywords:** machine learning, generalized regression neural networks, extreme learning machine, support vector regression, biocomposites

## Abstract

The growing demand for sustainable materials has significantly increased interest in biocomposites, which are made from renewable raw materials and have excellent mechanical properties. The use of machine learning (ML) can improve our understanding of their mechanical behavior while saving costs and time. In this study, the mechanical behavior of innovative biocomposite sandwich structures under quasi-static out-of-plane compression was investigated using ML algorithms to analyze the effects of geometric variations on load-bearing capacities. A comprehensive dataset of experimental mechanical tests focusing on compression loading was employed, evaluating three ML models—generalized regression neural networks (GRNN), extreme learning machine (ELM), and support vector regression (SVR). Performance indicators such as R-squared (R^2^), mean absolute error (MAE), and root mean square error (RMSE) were used to compare the models. It was shown that the GRNN model with an RMSE of 0.0301, an MAE of 0.0177, and R^2^ of 0.9999 in the training dataset, and an RMSE of 0.0874, MAE of 0.0489, and R^2^ of 0.9993 in the testing set had a higher predictive accuracy. In contrast, the ELM model showed moderate performance, while the SVR model had the lowest accuracy with RMSE, MAE, and R^2^ values of 0.5769, 0.3782, and 0.9700 for training, and RMSE, MAE, and R^2^ values of 0.5980, 0.3976 and 0.9695 for testing, suggesting that it has limited effectiveness in predicting the mechanical behavior of the biocomposite structures. The nonlinear load-displacement behavior, including critical peaks and fluctuations, was effectively captured by the GRNN model for both the training and test datasets. The progressive improvement in model performance from SVR to ELM to GRNN was illustrated, highlighting the increasing complexity and capability of machine learning models in capturing detailed nonlinear relationships. The superior performance and generalization ability of the GRNN model were confirmed by the Taylor diagram and Williams plot, with the majority of testing samples falling within the applicability domain, indicating strong generalization to new, unseen data. The results demonstrate the potential of using advanced ML models to accurately predict the mechanical behavior of biocomposites, enabling more efficient and cost-effective development and optimization processes in the field of sustainable materials.

## 1. Introduction

Composite sandwich panels are pivotal across various industries and renowned for their outstanding mechanical properties, such as a high strength-to-weight ratio and exceptional rigidity. These panels find critical applications in the aerospace, automotive, marine, and construction sectors. Extensive research has explored their mechanical behavior through experimental, numerical, and theoretical analyses [1,2,3,4]. However, conventional methods often fall short in accurately modeling the intricate interactions within composite materials. This gap has been addressed by the advent of data science and machine learning (ML) algorithms, which have revolutionized the study and prediction of the mechanical responses of composite components [5,6]. By leveraging extensive datasets and sophisticated algorithms, researchers can uncover hidden patterns and correlations, enhancing our understanding and predictive capabilities in the development and optimization of composite materials. This paper investigates recent advancements in ML applications, underscoring their transformative role in predicting mechanical properties and optimizing the design of composite structures for superior performance [7,8]. Despite significant progress, understanding and predicting the mechanical characteristics of these materials remain challenging due to their complexity. To address these challenges, researchers have progressively incorporated data science and ML techniques, utilizing vast datasets and computational resources to deepen our knowledge of the mechanical properties of composite materials. For instance, Yuan et al. [9] investigated the mechanical properties of rubber used in shock absorbers for landing gear in new energy electric aircraft. Their integration of artificial neural networks (ANNs) with self-adaptive particle swarm optimization demonstrated superior prediction accuracy, highlighting the potential of ML in constitutive modeling. Similarly, Wang et al. [10] tackled the challenges of predicting the mechanical properties of braided textile composites, showing the potential of ANNs-based feedforward backpropagation (FFBP) algorithms in supporting the design of composites for enhanced energy absorption. Zhang et al. [11] explored the mechanical properties of composites made from polypropylene (PP) and waste ground rubber tire powder (WGRT), optimizing the formulations using a hybrid ANN–genetic algorithm technique, which underscored the effectiveness of ML-driven approaches in commercial composite formulation. In damage prediction, Osa-uwagboe et al. [12] proposed an ML model to predict damage behavior in E-glass fiber-reinforced plastics under out-of-plane loading, utilizing regression algorithms and acoustic signal monitoring to develop an autonomous optimization model for composite structures. Viotti and Gomes [13] identified 3D delamination in sandwich composite structures using ML to detect and parameterize damage, emphasizing the importance of automated structural health monitoring techniques. Material optimization was further addressed by Takagi et al. [14], who proposed an ML-based approach to optimize the geometric arrangement of cellular materials to enhance mechanical performance, demonstrating accurate predictions and the benefits of ML in material development. Singh et al. [15] integrated finite element (FE) simulations and a genetic algorithm (GA) to optimize braided beam structures within the spaceframe chassis of rail vehicles, showcasing the advantages of combining FE simulations and GA for component-level optimization while ensuring overall structural performance. Zhang et al. [16] developed a computational framework integrating ML and multi-objective optimization for designing space-deployable bistable composite structures, highlighting the significant influence of ply angle on structural behavior. Li et al. [17] developed an interpretable ML model to elucidate the relationship between microstructure and properties of UD-CFRP composites with microvoids, demonstrating the effectiveness of materials informatics in understanding structure–property relationships. Zhao et al. [18] introduced a highly efficient ML-based multiscale modeling strategy for predicting CFRP properties, promising to accelerate composite material design and development cycles. Li et al. [19] investigated thermal activation effects on cement paste and utilized ML techniques to predict the compressive strength of TA-modified cement mortar, offering insights into optimal curing conditions for cement-based materials. Zhang et al. [20] analyzed foam concrete samples using ML models to predict compressive strength, providing valuable insights for construction applications. Wang et al. [21] examined hardness variations in multilayer composites through experimental methods and ML, demonstrating accurate predictions and applicability to other systems. Ferdousi et al. [22] explored 3D printable lightweight hybrid composites, showing the potential for predictive modeling using conventional statistics and convolutional neural network (CNN)-driven image analysis. Jiang et al. [23] introduced a multi-objective optimization method using CNNs to streamline the design of digital composite structures, enabling unique properties such as shape morphing and programmable deformation. Sharma et al. [24] employed ML to develop a novel PEEK composite with improved tribological and mechanical properties for biomedical applications. Hao et al. [25] proposed a multi-instance multi-label framework to evaluate ultimate tensile strength and the relationships between microstructure and properties in ternary metal composites, integrating finite element simulations and ML techniques. Karathanasopoulos et al. [26] investigated displacement and stress fields in orthotropic composite beams with variable stiffness using ML and explainable artificial intelligence, emphasizing the nonlinear effects of orthotropy and material gradation on mechanical response. These studies collectively highlight the transformative potential of ML and data science in advancing materials science and engineering. Through computational tools and extensive data sets, researchers are driving the design and optimization of materials, leading to innovation in various industries. In this research, ML algorithms are used to study the mechanical behavior of biocomposite sandwich structures under quasi-static out-of-plane compression. The predictive capability of ML offers significant advantages: Predicting new sample results without physical testing saves time and resources; accurate predictions enable rapid design iterations and material formulations, accelerating development cycles; and less physical testing reduces testing costs. Consequently, ML models efficiently analyze the effects of geometric variations on load-bearing capacity, streamlining the research and development process and demonstrating their critical role in optimizing biocomposite sandwich structures. Specifically, in this research, we utilize generalized regression neural networks (GRNN), extreme learning machines (ELM), and support vector regression (SVR) to develop predictive models. The performance of these models is evaluated using a range of metrics to ensure robustness and reliability. Furthermore, advanced visualization tools such as Taylor diagrams and Williams plots are employed to assess model accuracy and generalization ability. These tools effectively capture the non-linearity of the load-displacement behavior inherent in bio-based cellular composite sandwich structures, providing a comprehensive understanding of their mechanical performance.

## 2. Experimental Methodology

**Figure 1:**
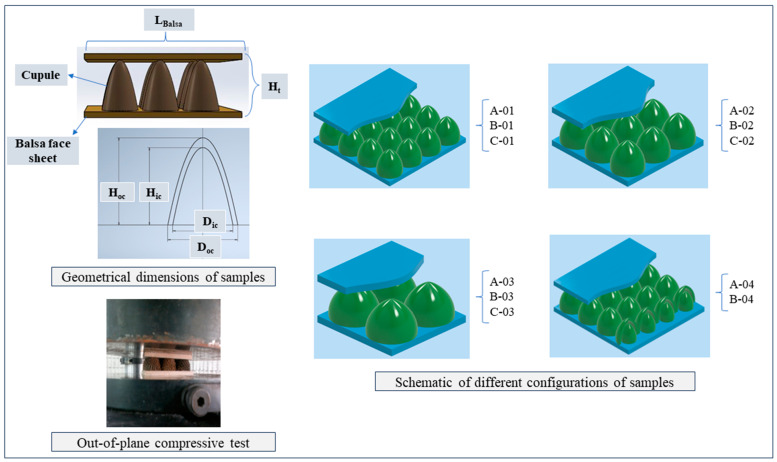
Manufacturing and compressive testing of sandwich panels (data utilized in this ML investigation).

This study employs machine learning techniques to forecast the mechanical properties of environmentally friendly composite panels subjected to quasi-static out-of-plane compression. The database utilized in this research is derived from the author’s previously published works [27,28]. Based on this, bio-based sandwich structures were prepared and tested, with oak tree cupules (OTCs) bonded onto balsa wood and categorized by geometric characteristics such as height, thickness, and diameter. Different core configurations with varying numbers of OTCs were also examined. Experimental methods assessed the compressive strength influenced by these geometric parameters, and the resulting data was analyzed to predict the compressive performance of the structures. Figure 1 depicts the manufactured sample, panel features, the quasi-static out-of-plane testing, and schematic diagrams of various core configurations. The geometrical characteristics for each specimen shown in Figure 1 are detailed in Table 1. In Table 1, the parameters n, H_c_, t_c_, D_c_, H_t_, and M_t_ denote critical dimensions and quantities pertinent to the biocomposite sandwich structures under investigation. Specifically, n indicates the number of cupules, H_c_ represents the outer height of the cupules, t_c_ refers to the thickness of the cupules, and D_c_ specifies the outer diameter of the cupules. Additionally, H_t_ denotes the total height of the sandwich panel, while M_t_ corresponds to the total weight of the specimens.

## 3. Workflow of ML Analysis

**Figure 2:**
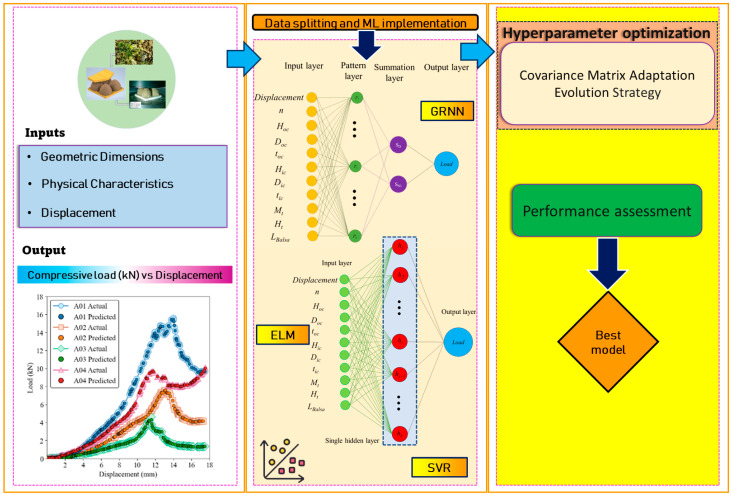
Workflow of utilizing ML to predict the mechanical behavior of bio-based sandwich structures.

Figure 2 illustrates the workflow employed in this study. Initially, the experimental test data was segmented into training and test datasets. Then, three ML algorithms were applied to predict the compressive load. Subsequent analysis included capturing the nonlinear load-displacement behavior and using Taylor diagrams and Williams plots to evaluate model accuracy and generalization ability.

### 3.1. Database Overview

Table 2 provides statistical summaries of key parameters for composite sandwich panels used in the ML analysis. It contains measured values such as the displacement (mm), the number of cupules (n), the height of the outer cupule (H_oc_), the diameter of the outer cupule (D_oc_), the thickness of the outer cupule (t_oc_), the height of inner cupule (H_ic_), the diameter of the inner cupule (D_ic_), the thickness of the inner cupule (t_ic_), the weight of the sandwich panel (M_t_, in grams), the total height of the panel (H_t_), the length of the facing layer (L_Balsa_, in mm), and the compressive load (kN). Each parameter is summarized with counts (2122 samples), means, standard deviations, and ranges (minimum to maximum). For example, the average displacement was 7.7 mm with a standard deviation of 4.7 mm. The number of cupules ranged from 4 to 16. The outer cupule’s height and diameter averaged 26.5 mm and 18.2 mm, respectively. The weight and compressive load showed considerable variability, averaging 24.1 g and 3.3 kN, respectively. This summary highlights the diversity in panel characteristics, which is essential for robust ML model training and performance evaluation.

**Figure 3:**
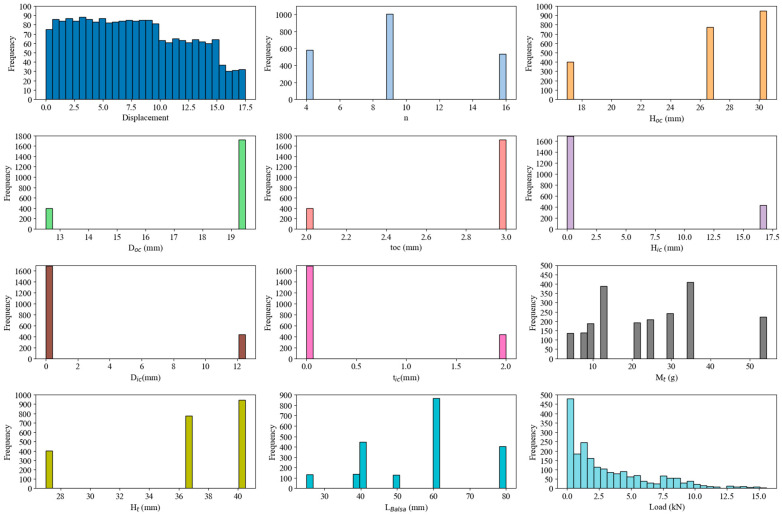
Frequency distributions of various parameters.

Figure 3 illustrates the frequency distributions of the various parameters in the database. For the displacement, the figure shows a relatively uniform distribution with a slight decrease in frequency at higher values. The histograms for n and H_oc_ indicate a few distinct values with the highest frequencies, indicating specific standard dimensions used in most panels. The histograms for D_oc_ and H_ic_ show that the majority of samples have values clustered at the higher end of their respective ranges. For D_ic_ and t_oc_, prominent peaks indicate that most panels have a minimum inner cupule diameter and moderate thickness. The histogram for M_t_ has a broad distribution of panel masses, with several peaks indicating different construction specifications. The histograms for H_t_ and L_Balsa_ histograms show clear clusters at certain heights and lengths, indicating standardized sizes. Finally, the load histogram shows a right-skewed distribution, indicating that most panels can withstand lower compressive loads, with fewer panels enduring higher loads. These distributions help to understand the common geometric configurations and mechanical properties of composite sandwich panels and provide important information for ML model training and prediction.

### 3.2. Model Development

#### 3.2.1. Generalized Regression Neural Network

**Figure 4:**
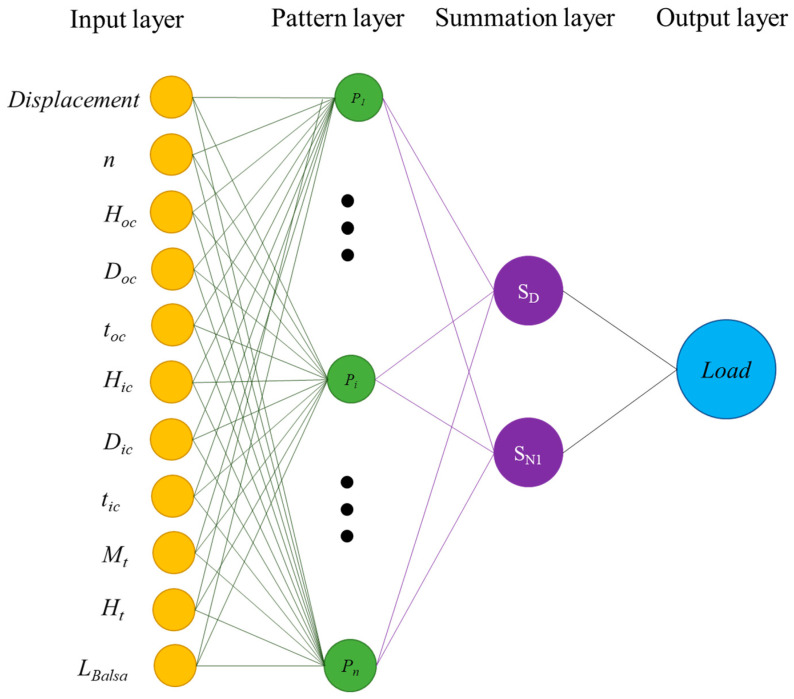
GRNN architecture.

Generalized regression neural networks (GRNN) are a type of artificial neural network used mainly for function approximation and prediction tasks. GRNNs are based on kernel regression techniques and use a radial basis function (RBF) network structure [29,30]. The structure of a generalized regression neural network (GRNN) comprises four layers: input layer, pattern layer, summation layer, and output layer (see Figure 4). The main feature of GRNNs is that they do not require iterative training, making them faster to train compared to traditional backpropagation neural networks. The RBF used in GRNNs is typically Gaussian, defined by the following formula:

(1) $$\varphi (x)=exp\left(-\frac{\left\|x-\mu \right\|}{2\sigma^{2}}\right)$$

where μ is the center of the RBF, σ is the spread parameter, and x is the input vector. GRNNs are particularly effective in modeling nonlinear systems and processing noisy data due to their robustness.

#### 3.2.2. Extreme Learning Machine

**Figure 5:**
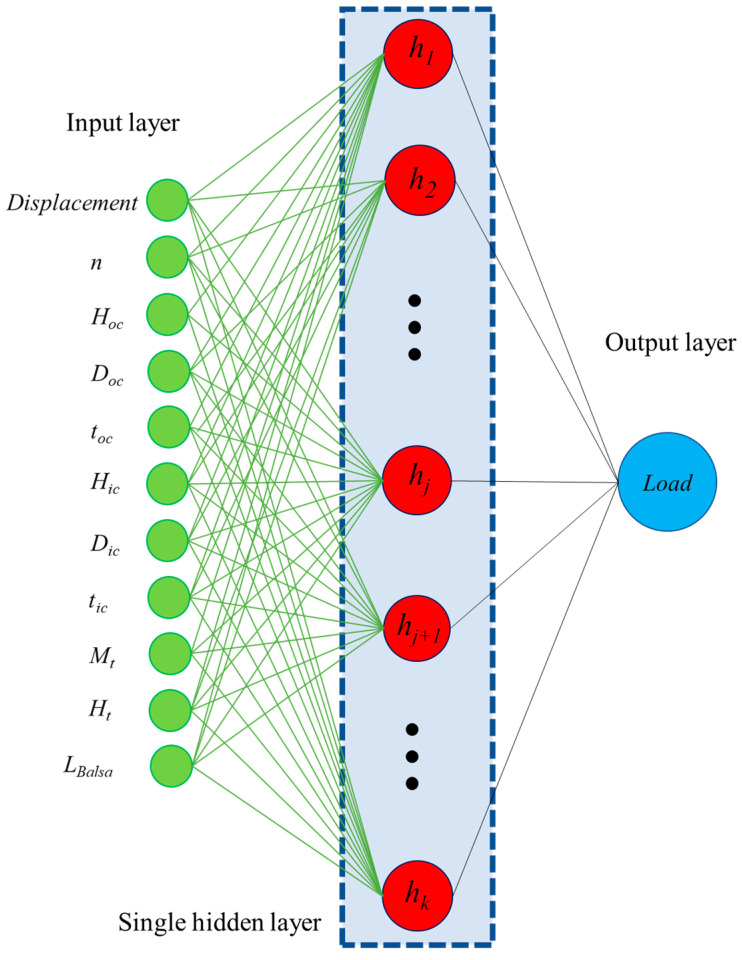
ELM architecture.

The learning algorithm known as extreme learning machine (ELM) is specifically crafted for single hidden layer feedforward neural networks. The architecture of an ELM includes an input layer, a single hidden layer, and an output layer (as illustrated in Figure 5). In contrast to conventional neural network training techniques, weights and biases for the hidden layer neurons in ELM are randomly allocated, and the output weights are computed analytically. This approach significantly reduces the training time and ensures good generalization performance. ELM’s key advantage is its speed and simplicity The ELM algorithm is known for its efficiency in training and robustness in handling different types of data [31,32,33].

#### 3.2.3. Support Vector Regression

Support vector regression (SVR) extends the capabilities of support vector machines to handle regression tasks. In the architecture of SVR, a kernel function is employed to transform input data into a higher-dimensional space where a linear regression model can be formulated. SVR operates by identifying a hyperplane that optimally fits the data while preserving a margin of tolerance defined by a parameter ϵ. This process relies on support vectors, a subset of the training data, to delineate this hyperplane [34,35]. The mathematical formulation of SVR aims to minimize the following objective function:

(2) $${min}_{w,b,\xi ,\xi *}\frac{1}{2}{\left\|w\right\|}^{2}+C\sum_{i=1}^{n}\left(\xi_{i}+\xi_{i}^{*}\right)$$

subject to:

(3) $$y_{i}-\left(w\cdot \varphi \left(x_{i}\right)+b\right)\leq \varepsilon +\xi_{i}$$

(4) $$(w\cdot \varphi (x_{i})+b)-y_{i}\leq \epsilon +\xi_{i}^{*}$$

(5) $$\xi_{i},\xi_{i}^{*}\geq 0$$

The kernel function is denoted as φ(x), while w and b represent the model parameters. Slack variables $\xi_{i}$ and $\xi_{i}^{*}$ are introduced, and C serves as a regularization parameter governing the balance between the regression function’s smoothness and the extent to which deviations exceeding ε are permissible.

### 3.3. Hyperparameter Tuning and Optimization

In this research, the covariance matrix adaptation evolution strategy (CMA-ES) was used to fine-tune hyperparameters for ML algorithms. The idea behind CMA-ES is based on evolutionary algorithms, which mimic the process of natural selection to solve complex optimization problems [36]. CMA-ES is particularly adept at dealing with high-dimensional, non-linear, and multimodal optimization problems, making it a powerful tool for hyperparameter tuning. Unlike conventional grid search or random search methods, CMA-ES adapts the covariance matrix of a multivariate normal distribution to sample candidate solutions, thereby efficiently exploring and exploiting the search space. This adaptive mechanism allows CMA-ES to focus on promising regions of the search space and converge more effectively to optimal or near-optimal solutions more effectively. By applying CMA-ES to fine-tune the hyperparameters of GRNN, ELM, and SVR, we were able to systematically adjust the parameters to enhance their predictive performance. This approach led to significant improvements in the accuracy and generalization capabilities of these models and proves the effectiveness of CMA-ES in optimizing ML algorithms for various regression tasks.

### 3.4. ML Evaluation Metrics

To assess and compare the performance of each algorithm, three metric measurements were employed: R-squared (R^2^), mean absolute error (MAE), and root mean square error (RMSE). R^2^ gauges the extent to which the regression predictions approximate the actual data points, indicating the proportion of variance in the dependent variable that can be predicted by the independent variables. Meanwhile, MAE quantifies the average magnitude of errors in a set of predictions, irrespective of their direction, providing insight into the absolute disparities between predicted and actual values. In contrast, RMSE offers a more sensitive evaluation of larger errors by first squaring the differences before averaging them and then taking the square root, thereby offering a more comprehensive perspective on the model’s predictive accuracy. By employing these metrics, we ensured a thorough and robust evaluation and comparison of the performance of the GRNN, ELM, and SVR models regarding their predictive capabilities. The equations for the three performance factors are explained below:

(6) $$R^{2}=1-\frac{\sum_{i=1}^{n}{\left({Load}_{i}-{Load}_{i}^{*}\right)}^{2}}{\sum_{i=1}^{n}{\left({Load}_{i}^{*}-\bar{Load}\right)}^{2}}$$

(7) $$RMSE=\sqrt{\frac{1}{n}\sum_{i=1}^{n}{\left({Load}_{i}-{Load}_{i}^{*}\right)}^{2}}$$

(8) $$MAE=\frac{1}{n}\sum_{i=1}^{n}\left|{Load}_{i}-{Load}_{i}^{*}\right|$$

where n is the number of experimental data points, ${Load}_{i}$ represents the predicted load values, ${Load}_{i}^{*}$ indicates the measured load values, and $\bar{Load}$ is the average load value.

## 4. Results and Discussion

In this study, we utilized three different ML algorithms to predict the load-displacement behavior of biocomposite oak sandwich panels. We split the data into 80% for training and 20% for testing. Using CMA-ES, we fine-tuned the algorithms, and the best hyperparameters are listed in Table 3. The evaluation of algorithms, summarized in Table 4, demonstrates that the GRNN model outperforms both the ELM and SVR models. The GRNN model showed superior performance with an RMSE of 0.0301, an MAE of 0.0177, and an R^2^ of 0.9999 in the training set, and an RMSE of 0.0874, an MAE of 0.0489, and an R^2^ of 0.9993 in the testing set, indicating near-perfect predictive accuracy and generalizability. The ELM model also performed well with RMSE, MAE, and R^2^ values for training of 0.2428, 0.1690, and 0.9946, and RMSE, MAE, and R^2^ values for testing of 0.2637, 0.1810, and 0.9940, showing slightly higher error rates and lower R^2^ values compared to GRNN, suggesting that it is robust but slightly less powerful. The SVR model had the lowest performance, with training RMSE, MAE, and R^2^ values of 0.5769, 0.3782, and 0.9700, and test RMSE, MAE, and R^2^ values of 0.5980, 0.3976, and 0.9695, indicating that it is less accurate and reliable than GRNN and ELM. The comparative analysis highlights that GRNN is the most accurate and reliable model for this application, followed by ELM, with SVR being the least effective. The near-perfect R^2^ values for the GRNN model suggest it can capture the intricacies of the material behavior very well, making it the preferred choice for predicting the load-displacement behavior of biocomposite sandwich panels made from oak.

### 4.1. Load-Displacement Behavior Prediction

**Figure 6:**
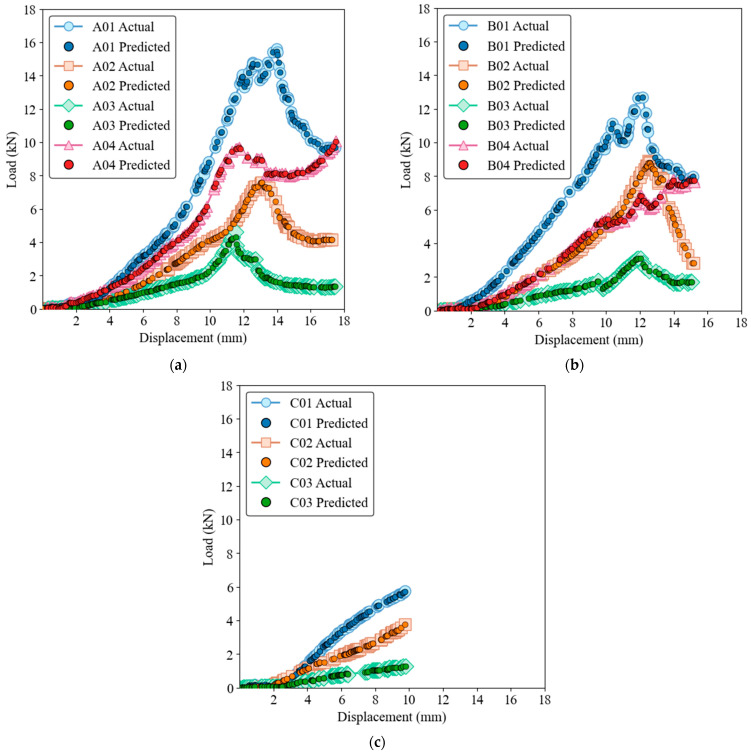
Load-displacement behavior for actual and GRNN predictions on the training dataset of ample groups A (**a**), B (**b**), and C (**c**).

**Figure 7:**
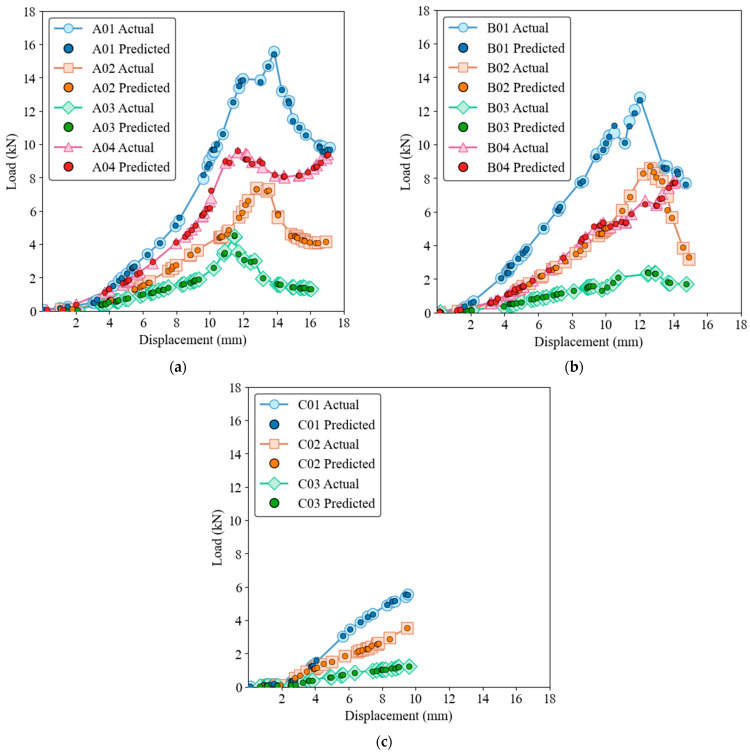
Load-displacement behavior for actual and GRNN predictions on the test dataset. Samples groups (**a**) A, (**b**) B, (**c**) C.

**Figure 8:**
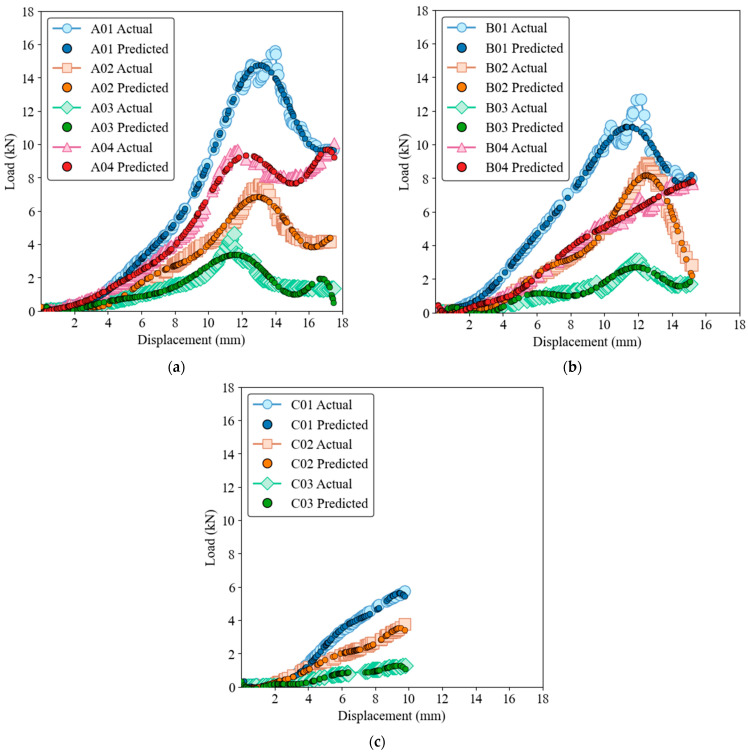
Load-displacement behavior for actual and ELM predictions on the training dataset. (**a**) Group A, (**b**) Group B, (**c**) Group C.

**Figure 9:**
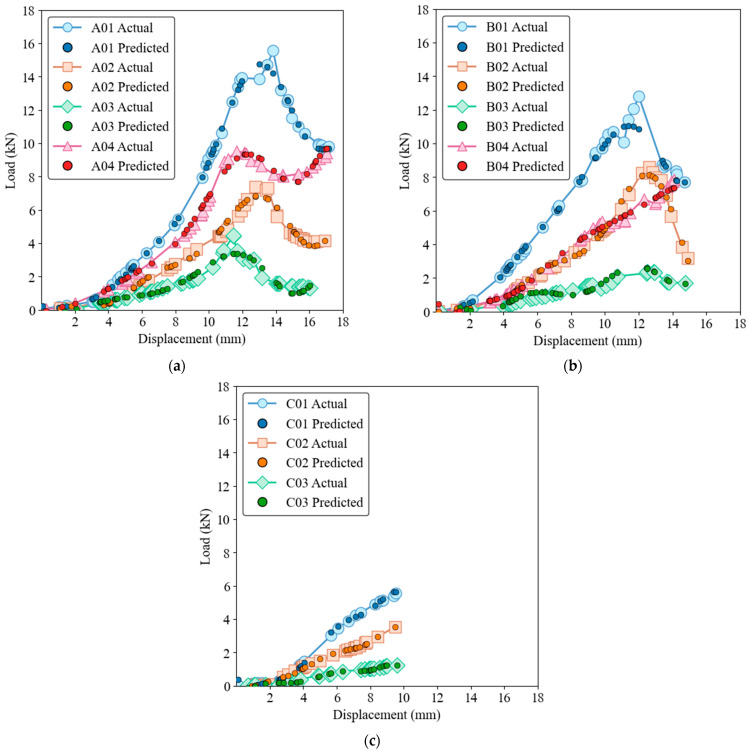
Load-displacement behavior for actual and ELM predictions on the test dataset. (**a**) Group A, (**b**) Group B, (**c**) Group C.

**Figure 10:**
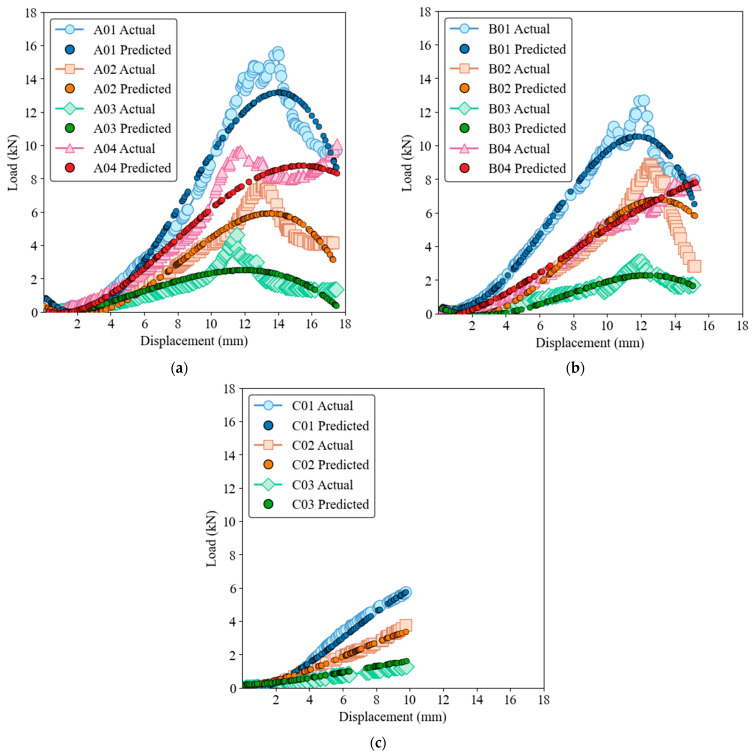
Load-displacement behavior for actual and SVR predictions on the training dataset. (**a**) Group A, (**b**) Group B, (**c**) Group C.

The capability of the three models to predict load-displacement behavior is illustrated in load-displacement Figure 6, Figure 7, Figure 8, Figure 9 and Figure 10 for both training and test sets. Figure 6 and Figure 7 show that the GRNN model effectively captures the nonlinear load-displacement behavior, including the peaks and fluctuations that represent critical points in the structural performance of the biocomposite panels, for both training and test datasets. GRNN’s architecture, which includes a highly adaptive neural network structure, allows it to handle complex and nonlinear relationships. This is reflected in its near-perfect predictions. The model accurately predicts the sharp increases in load followed by peaks and subsequent drops, which is essential for understanding the failure points and load-bearing capacity of the material. This ability is especially pronounced in nonlinear regions where other models struggle.

Figure 8 and Figure 9 show that while the ELM model has a robust ability to capture general trends and patterns, it also has some deviations, especially around the peak load points, for both training and test datasets. The performance of the ELM model is consistent, following the initial elastic region and nonlinear behavior up to the peak load, with slight discrepancies in the peak and post-peak region. Despite these discrepancies, the ELM model remains a reliable option for predicting load-displacement behavior. Due to its simpler architecture compared to GRNN, it is less computationally intensive, although this comes at the cost of some precision in capturing complex behaviors.

**Figure 11:**
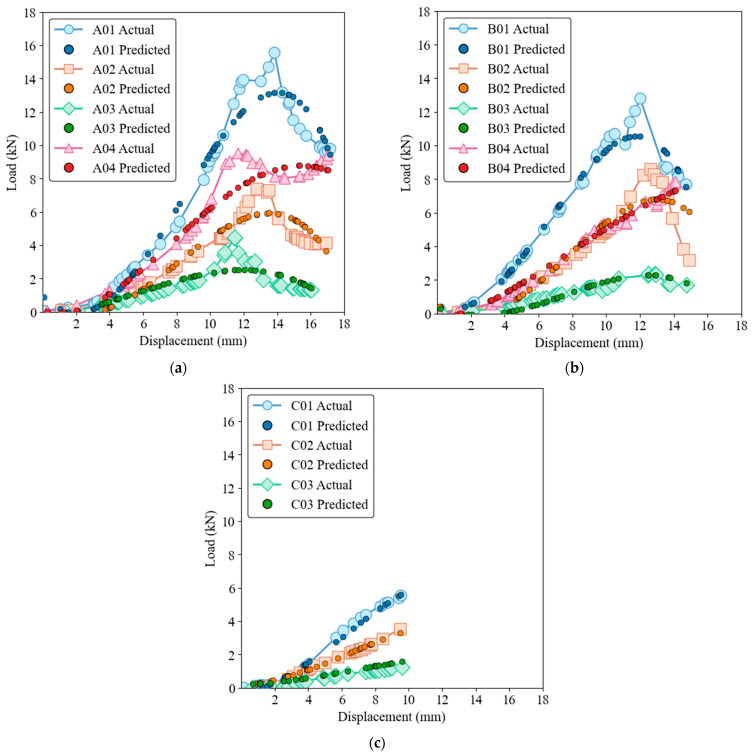
Load-displacement behavior for actual and SVR predictions on the test dataset. (**a**) Group A, (**b**) Group B, (**c**) Group C.

Figure 10 and Figure 11 for SVR show that although the SVR model provides a reasonable approximation of load-displacement behavior, it has lower precision compared to GRNN and ELM models for both the training and test datasets. The SVR model captures the general trend and slope of the load-displacement curves but struggles to accurately predict sharp peaks and subsequent drops, which are critical to understanding the structural performance of the material. The deviations are more pronounced than with the ELM model, highlighting the limitation of SVR in accuracy at critical points.

The samples of group C, which shows a rather linear behavior, can be predicted well with all three models. The linear relationship between load and displacement is simple for these specimens and represents only minimal complexity. However, when it comes to nonlinear behavior and complex upwings and downwings, the GRNN model excels in its ability to effectively capture complicated patterns and nonlinear relationships. The ELM model also performs well but has slight deviations at critical points, while the SVR model, although reasonable, has the highest degree of inaccuracy among the three models. The complexity of the GRNN model’s architecture, which includes a highly adaptive neural network structure, allows it to handle more complex relationships in the data compared to the simpler architectures of ELM and SVR. The ability of GRNN to generalize from the training data to new, unseen data makes it the preferred model for applications that require high precision. In contrast, ELM and SVR, while easier to implement and less computationally intensive, are better suited for applications where general trends are sufficient and extreme precision is not critical.

From an evolutionary perspective, these models can be seen as progressively advanced versions of each other. SVR, a traditional ML method, provides a basis for understanding linear and some nonlinear relationships. ELM introduces a single-layer feedforward neural network that improves the model’s ability to learn more complex patterns with shorter training times. GRNN, the most advanced of the three models, uses a more sophisticated neural network architecture that can capture highly nonlinear and complex behavior and shows superior performance in both the training and testing phases. The figures highlight the superior performance of the GRNN model in both the training and testing phases and illustrate its robustness and accuracy in modeling the complex load-displacement behavior of oak biocomposite sandwich panels. This development from SVR to ELM to GRNN illustrates the increasing complexity and ability of ML models to capture detailed and nonlinear relationships in data.

### 4.2. Prediction of Load-Displacement Behavior

**Figure 12:**
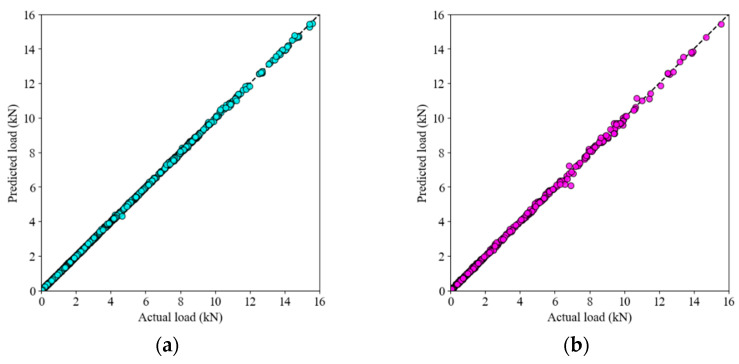

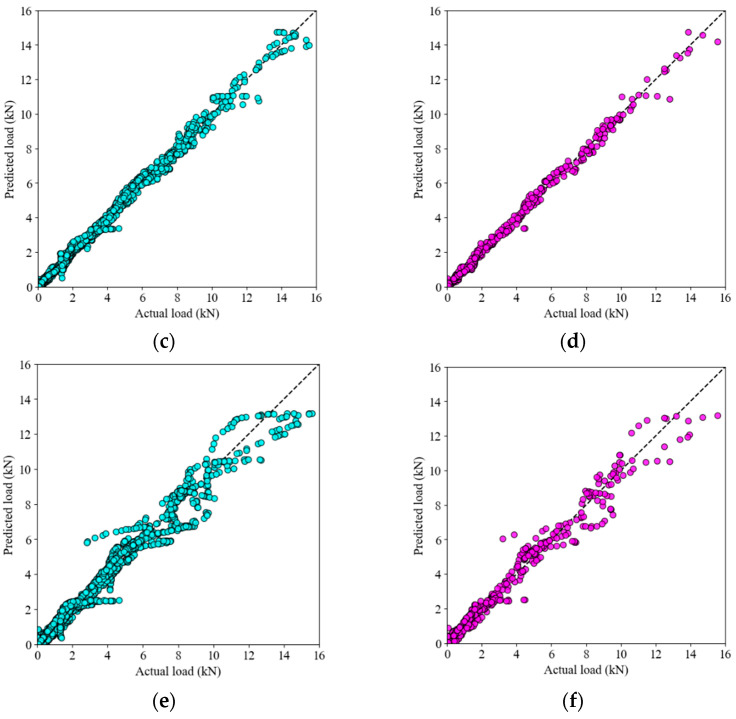
Cross-plots for (**a**) GRNN (train), (**b**) GRNN (test), (**c**) ELM (train), (**d**) ELM (test), (**e**) SVR (train), and (**f**) SVR (test).

The cross plots illustrate the predictive capabilities of the GRNN, ELM, and SVR models by comparing the actual and predicted load values for both training and test datasets. In Figure 12a,b, the GRNN model shows outstanding performance, with the predicted load values closely matching the actual values for both the training and test datasets. Figure 12c,d shows the performance of the ELM model. While it accurately predicts the load values for the training data, the scatter is somewhat more pronounced compared to GRNN. The test data shows larger deviations from the diagonal line, indicating a decrease in accuracy. Despite these minor discrepancies, ELM remains a strong performer, albeit not as robust as GRNN, especially in more complicated load-displacement scenarios. Figure 12e,f illustrates the results of the SVR model. The training data shows a reasonable fit, but the deviations from the diagonal line are more pronounced. The test data reveals even greater scatter, highlighting the difficulties of SVR in generalizing and accurately capturing the complex, nonlinear behavior of the load-displacement relationship.

**Figure 13:**
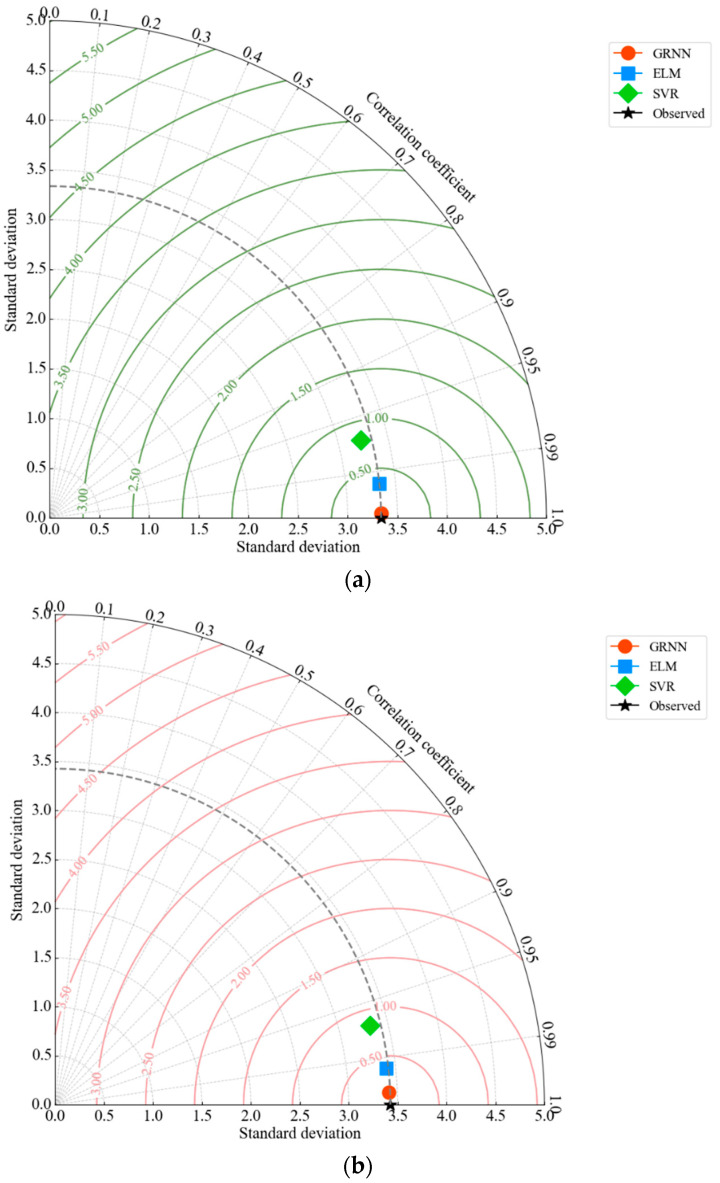
Taylor diagram for (**a**) training dataset, (**b**) testing dataset.

A Taylor diagram is a powerful graphical tool for evaluating the performance of predictive models. It displays three statistics simultaneously: The R^2^ value (which indicates the accuracy of the prediction), the standard deviation (which indicates the dispersion or variability), and the RMSE (which reflects the difference between predicted and observed values). This comprehensive visualization enables an intuitive comparison of how well the different models replicate the observed data. For the training phase (Figure 13a), the Taylor diagram shows that the GRNN model (orange circle) is closest to the observed data point (black star). This proximity indicates that the GRNN model achieves the highest correlation and the most accurate standard deviation, reflecting excellent agreement with the training data. The ELM model (blue square) also performs well but with slightly lower accuracy compared to GRNN. The SVR model (green diamond) is furthest away from the observed data, indicating a lower correlation and a higher standard deviation. For the test data (Figure 13b), the Taylor diagram confirms that GRNN is closest to the observed data, demonstrating its superior generalization ability. Although the ELM model is still accurate, it shows a slight decrease in accuracy compared to GRNN. The SVR model shows the largest deviations, indicating the lowest predictive performance among the three models. These results highlight the superior ability of GRNN to model the load-displacement behavior of biocomposite sandwich panels, followed by ELM, with SVR showing the lowest accuracy. The Taylor diagram effectively illustrates the progressive improvement in model performance from SVR to ELM to GRNN.

**Figure 14:**
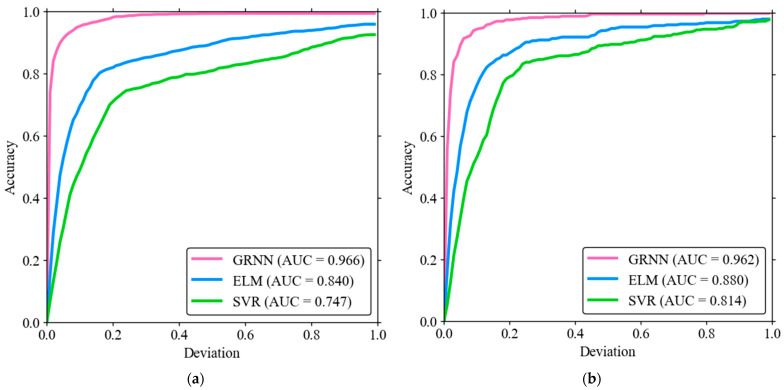
AUC analysis for (**a**) Training dataset, (**b**) Testing dataset.

The area under the curve (AUC) is a metric used for evaluating the performance of models and, while traditionally applied to classification models, it can also be adapted for regression models. In this context, AUC measures the area under the curve on a plot of accuracy versus deviation. A higher AUC value indicates better performance of the model, as it signifies a greater ability to predict continuous outcomes accurately. In the training phase (Figure 14a), the AUC values show the superior performance of the GRNN model with an AUC value of 0.966. This high value indicates excellent accuracy and predictive ability. The ELM model follows with a respectable AUC value of 0.880, showing good performance but not reaching the precision of GRNN. The SVR model has the lowest AUC at 0.814, reflecting its comparatively lower accuracy in the training phase. In the test dataset (Figure 14b), the AUC values further confirm the hierarchy observed in the training phase. GRNN achieves the highest AUC value of 0.962, underlining its robust generalization ability. The ELM model achieves good performance with an AUC value of 0.840, which is slightly lower than in the training phase, but still reliable. The SVR model continues to have the lowest accuracy with an AUC of 0.747, indicating its difficulty in generalizing to unseen data. These quantitative results of the AUC metrics highlight the better performance of the GRNN model in capturing the load-displacement behavior of biocomposite sandwich panels. The ELM model, while effective, falls short of the precision of GRNN, and the SVR model with the lowest AUC values shows the least effectiveness in both the training and testing phases. This comprehensive analysis confirms GRNN as the most accurate and reliable model for this application, followed by ELM and then SVR.

**Figure 15:**
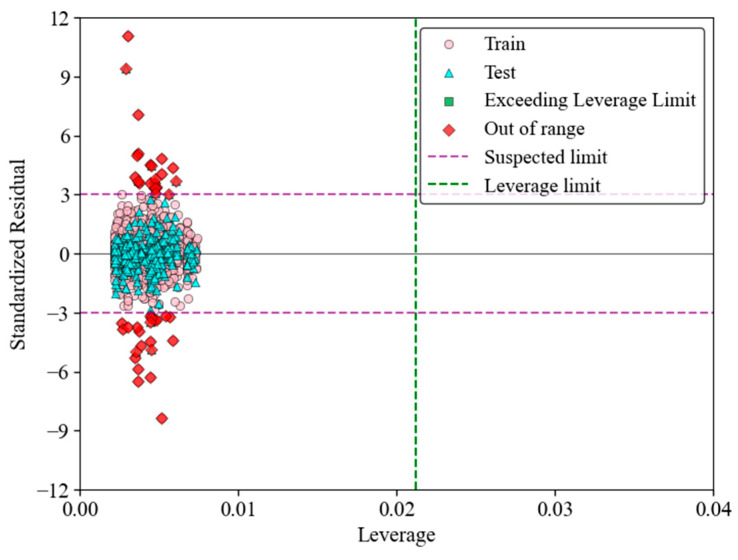
Williams plot illustrating the performance of GRNN.

A Williams plot is a diagnostic tool used to evaluate the applicability domain (AD) of a predictive model. It plots the leverage values on the x-axis against the standardized residuals on the y-axis. the leverage values show the influence of the individual data points on the model, while the standardized residuals reflect the prediction errors. Points that lie within the limits of ±3 standardized residuals and below a certain leverage threshold are considered to be within the AD of the model, which means that the prediction of the model for these points is reliable. In Figure 15, the Williams plot for the GRNN model shows the standardized residuals versus the leverage values for both the training and test datasets. The green dashed line represents the leverage limit, and the purple dashed lines indicate the boundaries for the standardized residuals. The plot shows that 98.05% of the training samples fall within the applicability domain. This high percentage demonstrates that the GRNN model has learned effectively from the training data, with most points showing low leverage and residuals within the acceptable range. The few points outside these limits indicate minimal outliers that have a negligible impact on the overall performance of the model. Similarly, 97.43% of the testing samples are within the applicability domain, indicating a strong generalization of the GRNN model to new, unseen data. The majority of the test data points also exhibit low leverage and standardized residuals within the acceptable range, confirming the reliability of the model’s predictions. The Williams plot highlights the superior performance of the GRNN model, with a high percentage of data points within the applicability domain for both the training and test datasets. This confirms the robustness and accuracy of the GRNN model in predicting the load-displacement behavior of biocomposite sandwich panels.

**Figure 16:**
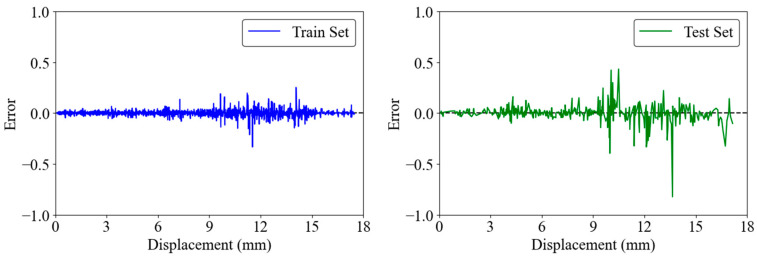
Detailed error analysis for the GRNN model.

Figure 16 shows the error between the predicted and actual load. In the training set, the errors are predominantly in the range of −0.5 to 0.5, indicating a high degree of accuracy and a good fit, as the errors are symmetrically distributed around zero, showing no significant bias. The test set exhibits a similar error range of −0.5 to 0.5, with slightly more variability compared to the training set, which could be attributed to the unseen data in the test set. These low and symmetric error ranges in both sets highlight the strong predictive performance and robust generalization capabilities of the model.

**Figure 17:**
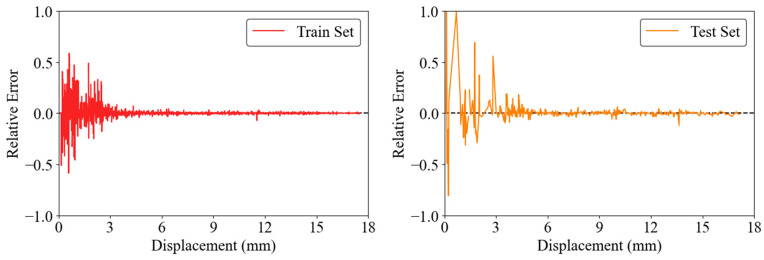
Relative error distribution for the GRNN model.

Figure 17 presents the relative error for the training set, showing that the relative errors are mainly in the range of −0.5 to 0.5, with the higher errors concentrated at lower displacements. This trend indicates that while the model performs well overall, its accuracy decreases when it predicts smaller displacements. In the test phase, the relative errors are also generally in the range of −0.5 to 0.5, with few peaks of up to 1.0, again mainly at smaller displacements. The decrease in relative error with increasing displacement shows that the accuracy of the model increases with larger displacements. Taken together, these plots confirm that most predictions are very accurate in both the training and testing phases, highlighting the effectiveness of the GRNN model in predicting load-displacement behavior. Despite some increase in error for the test set, the overall performance remains strong, highlighting the reliability and robustness of the GRNN model.

## 5. Conclusions

This study investigated the mechanical behavior of biocomposite sandwich structures under quasi-static out-of-plane compression using ML algorithms to predict load-displacement behavior. A comprehensive dataset of experimental mechanical tests focusing on compressive loading was used to evaluate three ML models: GRNN, ELM, and SVR, using performance metrics such as R^2^, MAE, and RMSE. The data was split 80% for training and 20% for testing, with the optimal hyperparameters for each model, determined using CMA-ES. The main results can be summarized as follows:

- The GRNN model showed a high predictive accuracy with a high R^2^ value, a low MAE and RMSE value, and a good generalization that effectively captures the complex nonlinear load-shift behavior.
- The SVR model was less powerful and showed less accurate predictions with a lower R^2^ value, a higher MAE and RMSE, and difficulties in predicting strong peaks and fluctuations.
- The ELM model captured general trends and patterns well but had some accuracy issues with load peaks, making it more suitable for general rather than detailed predictions.
- The Taylor diagrams showed that the GRNN model had the highest correlation and the most accurate standard deviation and therefore performed better than the other models.
- The GRNN model had the highest AUC values for both the training and test data, indicating better performance compared to the other models.
- The Williams plot indicated a high percentage of data points within the application range for the GRNN model, confirming its robustness and accuracy in predicting load-displacement behavior and its reliability for practical applications.

This study demonstrates the crucial role of ML in understanding and optimizing the mechanical behavior of biocomposites, resulting in lower costs, less time, and faster design and laboratory processes. Through the use of GRNN algorithms, the analysis of biocomposite sandwich structures under quasi-static compression can minimize the need for extensive physical testing and enable precise optimization of material properties.

## Figures and Tables

**Table 1:** Geometrical dimensions of specimens.

Specimen No.	n	H_c_ (mm)	t_c_ (mm)	D_c_ (mm)	H_t_ (mm)	M_t_ (g)	Cupule Type
A-01	16	30.5	3	19.5	40.5	54.29	Only A
A-02	9	30.5	3	19.5	40.5	29.81	Only A
A-03	4	30.5	3	19.5	40.5	13.47	Only A
A-04	9	30.5	3	19.5	40.5	34.89	A_outter_ + C_inner_
B-01	16	26.5	3	19.5	36.5	34.18	Only B
B-02	9	26.5	3	19.5	36.5	21.43	Only B
B-03	4	26.5	3	19.5	36.5	9.53	Only B
B-04	9	26.5	3	19.5	36.5	25.49	B_outter_ + C_inner_
C-01	16	17	2	12.5	27	12	Only C
C-02	9	17	2	12.5	27	7.92	Only C
C-03	4	17	2	12.5	27	3.47	Only C

**Table 2:** An overview of the database.

	Displacement (mm)	n	H_oc_ (mm)	D_oc_ (mm)	t_oc_ (mm)	H_ic_ (mm)	D_ic_ (mm)	t_ic_ (mm)	M_t_ (g)	H_t_ (mm)	L_Balsa_ (mm)	Load (kN)
count	2122	2122	2122	2122	2122	2122	2122	2122	2122	2122	2122	2122
mean	7.7	9.4	26.5	18.2	2.8	3.5	2.6	0.4	24.1	36.5	55.4	3.3
std	4.7	4.4	4.9	2.7	0.4	6.9	5.0	0.8	14.4	4.9	16.0	3.4
min	0.0	4.0	17.0	12.5	2.0	0.0	0.0	0.0	3.5	27.0	25.0	0.0
25%	3.7	4.0	26.5	19.5	3.0	0.0	0.0	0.0	12.0	36.5	40.0	0.6
50%	7.4	9.0	26.5	19.5	3.0	0.0	0.0	0.0	25.5	36.5	60.0	2.0
75%	11.4	16.0	30.5	19.5	3.0	0.0	0.0	0.0	34.2	40.5	80.0	5.1
max	17.5	16.0	30.5	19.5	3.0	17.0	12.5	2.0	54.3	40.5	80.0	15.6

**Table 3:** Best hyperparameters.

Model	Hyperparameter	Value
SVR	C	995.37
	ε	0.3591
ELM	Number of neurons	484
	Activation function	Tanh
GRNN	σ	0.00275

**Table 4:** Summary of evaluation.

Model	Training Dataset			Test Dataset		
	RMSE	MAE	R^2^	RMSE	MAE	R^2^
GRNN	0.0301	0.0177	0.9999	0.0874	0.0489	0.9993
ELM	0.2428	0.1690	0.9946	0.2637	0.1810	0.9940
SVR	0.5769	0.3782	0.9700	0.5980	0.3976	0.9695

## Data Availability

The original contributions presented in the study are included in the article, further inquiries can be directed to the corresponding author.
